# No evidence for disruption of reconsolidation of conditioned threat memories with a cognitively demanding intervention

**DOI:** 10.1038/s41598-022-10184-1

**Published:** 2022-04-22

**Authors:** Lars Jaswetz, Lycia D. de Voogd, Eni S. Becker, Karin Roelofs

**Affiliations:** 1 Donders Institute for Brain, Cognition and Behaviour, Radboud University, Nijmegen, The Netherlands; 2 Behavioural Science Institute, Radboud University, Nijmegen, The Netherlands

**Keywords:** Cognitive neuroscience, Amygdala, Prefrontal cortex, Psychology, Human behaviour

## Abstract

Simultaneous execution of memory retrieval and cognitively demanding interventions alter the subjective experience of aversive memories. This principle can be used in treatment to target traumatic memories. An often-used interpretation is that cognitive demand interferes with memory reconsolidation. Laboratory models applying this technique often do not meet some important procedural steps thought necessary to trigger reconsolidation. It remains therefore unclear whether cognitively demanding interventions can alter the reconsolidation process of aversive memories. Here, 78 (41 included) healthy participants completed an established 3-day threat conditioning paradigm. Two conditioned stimuli were paired with a shock (CS+ s) and one was not (CS-). The next day, one CS+ (CS+ R), but not the other (CS+), was presented as a reminder. After 10 min, participants performed a 2-back working memory task. On day three, we assessed retention. We found successful acquisition of conditioned threat and retention (CS+ s > CS-). However, SCRs to the CS+ R and the CS+ during retention did not significantly differ. Although threat conditioning was successful, the well-established cognitively demanding intervention did not alter the reconsolidation process of conditioned threat memories. These findings challenge current views on how cognitively demand may enhance psychotherapy-outcome.

## Introduction

Experimental studies consistently show that reactivation of aversive memories while simultaneously executing a cognitively demanding intervention can alter these reactivated memories such that they become less aversive when retrieved later (for a meta-analysis see^1^). This principle is used in eye-movement desensitisation and reprocessing therapy (EMDR^2^;) to target traumatic memories. A popular interpretation of this finding is that simultaneous execution of memory retrieval and a cognitively demanding intervention alters the *reconsolidation* process of the retrieved memory. Here, reconsolidation refers to a process in which a reminder^3^ can return a consolidated memory to a labile state after which the memory would require to be re-stabilised for the memory to be maintained^4–7^. Indeed, pharmacological interventions applied during this critical time period (i.e. up to 6 h after reactivation; e.g.^4,8,9^) were shown to disrupt the reconsolidation processing leading to impairment of the reactivated memory (^4^, but see^10,11^ for alternative accounts of these findings). However, cognitively demanding intervention studies often do not meet some important procedural steps thought necessary to destabilise the memory and trigger a reconsolidation process. Interestingly, it has been shown that the reconsolidation process of aversive memories can be impaired with other non-cognitively demanding tasks that induce new learning^12–14^. However, it remains unclear whether cognitively demanding interventions can alter the reconsolidation process of aversive memories.

Reconsolidation is an intricate process, which requires some procedural steps for the memory to destabilise and potentially be interfered with^15^. For example, animal studies have suggested that following memory reactivation, molecular processes such as protein synthesis, have to take place before the memory is fully destabilised^16–19^, similar to consolidation^20,21^. Therefore, it is possible that memory destabilisation does not happen instantaneously and that cognitively demanding interventions that occur *simultaneously* with memory reactivation therefore might not affect the reconsolidation process. Indeed, cognitively demanding intervention studies^22–26^ typically do not include a delay between the initial memory retrieval and the intervention for memory destabilisation to take place. These interventions might therefore not be able to interfere with the reconsolidation process. Furthermore, repeated reminders, as opposed to a single brief reminder, have been shown to trigger a process of extinction rather than reconsolidation^27^. Instead of altering the existing aversive memory, extinction leads to the formation of a new inhibitory memory^28^. In the intervention studies, the process of memory reactivation is typically repeated multiple times (e.g.^22–26^; and for a meta-analysis see^1^) thereby possibly inducing extinction. It is therefore possible that cognitively demanding interventions, instead of interfering with reconsolidation, may affect the process of extinction. Indeed, there is evidence showing that cognitively demanding interventions may enhance extinction learning in humans^29,30^ and mice^31^. Therefore, based on the procedure of the cognitively demanding intervention studies alone, it is not clear whether cognitively demanding interventions can alter the reconsolidation process of aversive memories.

Nevertheless, there is evidence suggesting it may be possible to alter the reconsolidation process of aversive memories with a cognitively demanding task. Namely, a few studies adhered to the above-mentioned procedural steps of which some have reported alterations of the reconsolidation process of aversive memories with a cognitively demanding intervention, while others have not. In two studies^32,33^, participants watched aversive movie clips, and after consolidation, at a later time point (1 day later^32^: or 3 days later^33^:), the memory of the movie clips was reactivated using still frames of the movie clips. After the memory reactivation procedure and a delay of 10 min, one group of participants performed a cognitively demanding computer game of Tetris with the goal to interfere with the reconsolidation of the memory for the movie clips. In both studies, the reactivation paired with cognitively demanding intervention led to significantly fewer intrusive memories of the aversive movie clips compared to the control conditions. A different study employed a similar design using a visuospatial stimulation task, which was not cognitively demanding^34^. Interestingly, they did not find a reduction in intrusions compared to a no-task control condition, implying that cognitive demand may be a crucial aspect in these paradigms. While these studies highlight the possibilities (and necessity) of cognitively demanding intervention in reducing intrusive memories, they are also limited to intrusive memories. It therefore remains the question whether cognitively demanding interventions can interfere with the reconsolidation of implicit memories such as conditioned threat.

A recent study investigated this by employing a threat conditioning paradigm^35^. Following memory reactivation of a conditioned stimulus (CS) that was previously associated with an aversive event (i.e. an electrical shock), one group of participants completed an emotional working memory task^36^. However, the authors did not find a significant difference in the conditioned response (i.e. measured with skin conductance and fear-potentiated startle) during retention between the intervention group and the control group that did not perform an emotional working memory task following memory reactivation. In contrast to earlier studies that typically utilized non-emotional tasks as an intervention (e.g.^1,22–26,32,33^), the authors^35^ instead made use of a cognitively demanding intervention with emotional pictures and neutral faces. As the presentation of emotional stimuli have been shown to increase amygdala activity^37–39^ it is possible that their task engaged the amygdala during the critical re-stabilization phase, instead of inhibiting it. Therefore, the emotional working memory task used by Chalkia and colleagues^35^ may not have interfered the reconsolidation process of the amygdala-dependent^40^ threat memory. This notion is important because animal models have shown that inhibiting the amygdala following memory reactivation, by blocking protein synthesis, disrupts the reconsolidation of threat memories^4^. In humans, the amygdala plays a vital role in several different emotional memory processes, such as consolidation and reconsolidation^41^. Furthermore, inhibition of the amygdala is a potential working mechanism through which cognitive demand could enhance extinction^29,42–44^. It remains therefore unknown whether a non-emotional working memory task, which has been shown to systematically inhibit the amygdala^43^, can disrupt the reconsolidation of conditioned threat memories (but see^45^ for a preprint).

Here, we tested the hypothesis that a cognitively demanding non-emotional working memory task after a brief memory reminder cue would interfere with the reconsolidation process of conditioned threat memories. In total 78 participants (41 included in the final sample) completed an established Pavlovian threat conditioning/reactivation/retention test paradigm across three consecutive days with a 24 h interval^46^. We used this paradigm as it enables direct comparison with human pharmacological studies, and because it helps animal-human translational interpretation, relevant because the reconsolidation literature is largely built on animal studies where threat conditioning is the golden standard. We predicted that reactivation of a conditioned stimulus (CS + R) followed by a cognitively demanding working memory task, within the reconsolidation window (10 min after reactivation), would lead to reduced conditioned threat responses during retention a day later, when compared to a conditioned stimulus that was not reactivated (CS +). Furthermore, we predicted that the CS + R would be resistant to reinstatement compared to the CS + , meaning that it would again evoke lower conditioned threat responses during a retention test after reinstatement. Finally, we expected that the CS + R would be rated as more likable compared to the CS + at the end of the experiment.

## Methods

This study was preregistered on the Open Science Framework before data collection (https://osf.io/dt8wr/). There were some minor deviations from the preregistration in relation to the exclusion criteria and statistical analyses. We explicitly indicate them in the paper. All research activities were approved by the local ethics committee (Ethical Reviewing Board CMO/METC [Institutional Research Review Board] Arnhem-Nijmegen, CMO 2014/288) and carried out in accordance with the Declaration of Helsinki.

## Participants

We recruited healthy individuals through the online recruitment system of the Radboud University. Inclusion criteria were: above the age of 18, with normal or corrected to normal vision, no acute mental disorder, no skin disease that would prohibit the use of electrodes, and no history of brain trauma or brain surgery. In total, 78 individuals (49 females, 29 males, 18–60 years [*M* = 24.73, *SD* = 7.05]) completed the entire study, with an additional three individuals who did not continue the study after the first or second day. From these 78 participants, 34 participants were excluded after completion of the study due to one or more of the following criteria: 1) mean SCR on one of the two CS + s being numerically smaller than the mean CS − response during the acquisition phase (one CS + lower than the CS-: *n* = 29, both CS + s lower than the CS-: *n* = 5, total: *n* = 34) or 2) a smaller average response than 0.05 μS in response to the unconditioned stimulus (UCS; *n* = 1, but this participant was also being excluded for having lower SCRs to one or both of the CS + s compared to the CS-). An additional three participants were excluded due to technical errors. The final sample therefore consisted of 41 participants (24 females, 17 males, 18–60 years [M = 25.27, SD = 8.30]) in line with the pre-registered sample size. According to an a-priori power analysis with G*Power^47^ for a repeated measures ANOVA design, a sample of 41 participants is sufficient to detect a small to medium effect size (f = 0.10–0.25, alpha level of 0.05, power level of 0.80). This sample size is sufficient to find an effect comparable to the effect found by Picco and colleagues^45^. All participants provided informed consent and received €16 as a compensation for their participation.

## Procedure

**Figure 1:**
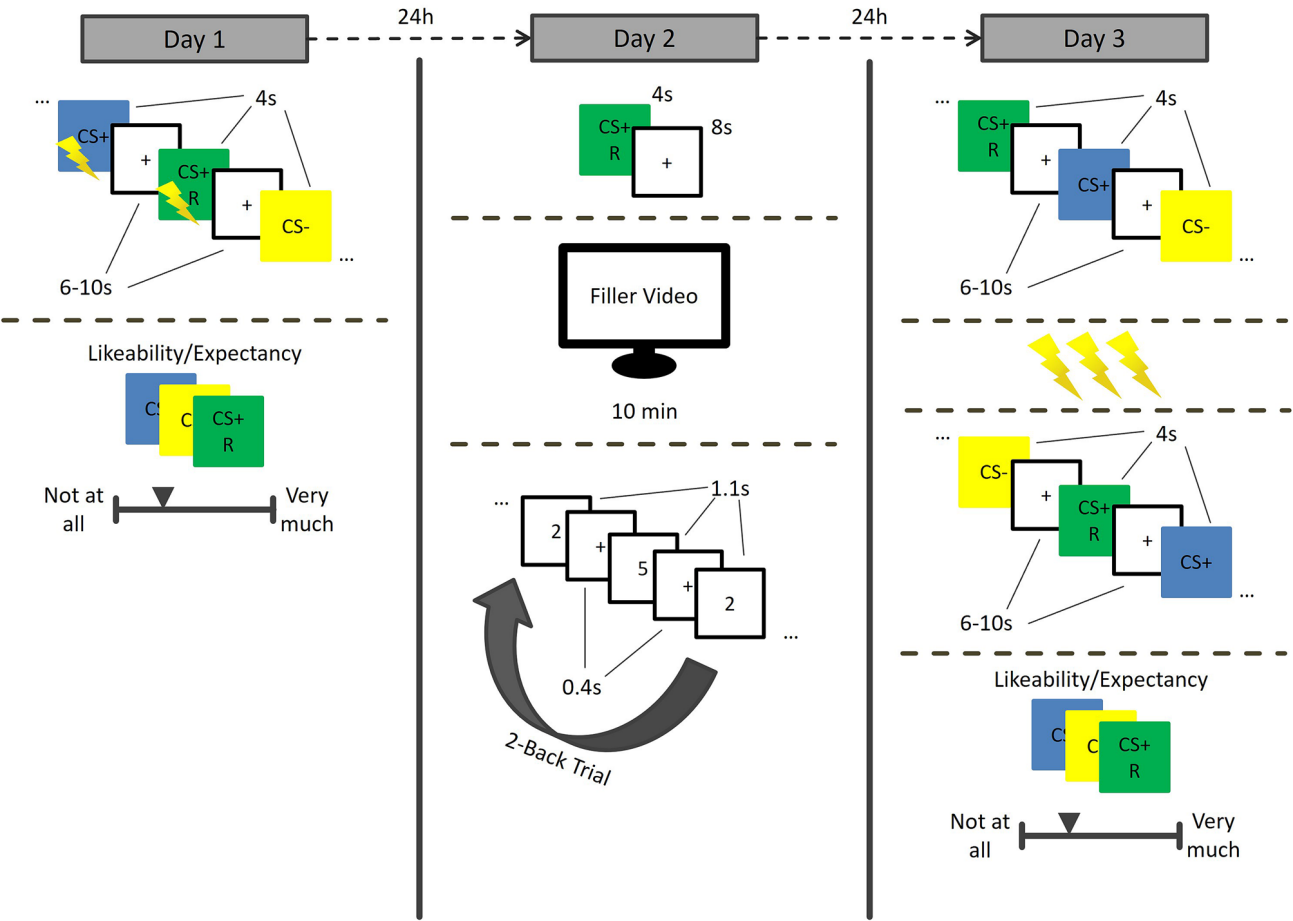
Schematic overview of the procedure per day. Day 1: Acquisition and subjective ratings. Day 2: Reactivation, 10 min filler video, 2-back working memory task. Day 3: Retention test, reinstatement, retention test after reinstatement. CS = conditioned stimulus, R = reactivated CS. This figure was created using Microsoft PowerPoint 2019 (https://www.microsoft.com/en-us/microsoft-365/powerpoint).

Participants were tested in a differential delay threat conditioning paradigm^46^ on three consecutive days with a 24 h interval in a within subject-design. For an overview, see Fig. 1. On the first day, participants came to the lab and filled in an informed consent for the entire study. On each day, participants were instructed to wash their hands to clean off any soap or disinfectant and, in case their hands were cold, to warm up their hands. Next, participants completed the shock workup procedure to calibrate the intensity of the electric shock (see below). Then, participants received instructions and practiced the 2-back working memory task, which they would perform the next day. The participants received written and schematic instructions of how the task worked (self-paced), followed by a demo of what the task looked like, and the practice block (both fixed pace). Afterwards, participants were subjected to the acquisition phase of threat conditioning paradigm. Finally, participants filled in a five-point rating scale concerning their shock expectancy and subjective feelings regarding the likeability of each stimulus. On the second day, participants completed the reactivation phase and 20 min of the 2-back working memory task. On the third day, participants completed the retention phase, including a retention test, a reinstatement procedure including 3 unsignaled shocks, and a retention test after reinstatement. Finally, participants again filled in the five-point rating scale concerning their shock expectancy and subjective feelings regarding the likeabiliy for each stimulus, similar to the first day. Afterwards, the participants were debriefed about the aim of the study.

## Material

### Conditioned stimuli

The conditioned stimuli (CS) consisted of three rectangles in the colours blue, green, and yellow. Two of these stimuli were used as CS + s and were paired with a shock, with one of the two serving as CS + R, which was reactivated on day 2. Additionally, one stimulus was used as a CS- and was never paired with a shock. Assignment of colours to the different CS types was counterbalanced across participants.

### Unconditioned stimulus

The unconditioned stimulus (UCS) was an electrical shock that was delivered via two Ag/AgCl electrodes attached to the distal phalanges of the second and third finger of the left hand. The shock was delivered via a MAXTENSE 2000 (Bio-Protech) electrical stimulation machine, with a frequency of 140 Hz and a duration of 200 ms. The shock intensity was set during a standardised shock workup procedure^29,48–50^ and remained the same throughout the whole experiment. In this procedure all participants received five shocks. After each shock, participants subjectively rated the experienced unpleasantness on a scale ranging from 1 (not painful at all) to 5 (very painful), based on which the subsequent shock was adjusted, in order to arrive at a shock intensity that was unpleasant, but not painful. The intensity varied in 10 intensity steps between 0 and 40 V/ 0–80 mA. The average intensity step was *M* = 4.45 (*SD* = 1.83).

### Differential delay threat conditioning paradigm

The threat conditioning paradigm included an acquisition phase, a reactivation phase, and a retention phase. Participants were instructed to discover the relationship between the CSs and the UCS.

The acquisition phase consisted of 16 stimulus presentation per CS type, resulting in 48 trials in total. The stimuli were presented in a pseudo randomised order, with no more than three stimuli of the same type being presented in succession. Each CS (4 s duration) was followed by an inter-trial interval (ITI) during which a fixation cross was shown (jittered 6–10 s, *M* = 8 s duration). For the CS + and the CS + R, the shock (200 ms duration) was delivered at 3.8 s after stimulus onset. Reinforcement rate was set at 37.5% for each CS + , meaning that six out of 16 CS + presentations were paired with a shock. The first presentation of each CS + was always paired with a shock to facilitate immediate and equal learning for both CS + types^29^. The remainder of the shocks were pseudo randomly distributed across the first and second half of the acquisition phase. This was done to ensure that the shocks were spread evenly across the whole acquisition phase.

During the reactivation phase, the CS + R was presented once (without a shock) for four seconds, followed by a fixed ITI of eight seconds. In order for the intervention to fall into the reconsolidation window, participants performed the 2-back working memory task after a ten-minute delay during which they watched a video clip depicting colourful fractals. This 10 min wait period is in line with other studies on reconsolidation^32,33,35,46,51^.

The retention phase consisted of a retention test, a reinstatement procedure, and a retention test after reinstatement. During the retention test, the participants were presented with all CSs but never received a shock. All CSs were shown with the same parameters as during the acquisition phase, except that each stimulus was shown 12 times. The first three trials of the retention test were always the three different CS types in an order that was counterbalanced across subjects. The reinstatement procedure consisted of three unsignaled shocks that were presented while the screen was turned black. The interval between the shocks was set to ten seconds, and the reinstatement procedure was preceded and followed by a ten second fixation cross presentation. During the retention after reinstatement test after the reinstatement procedure, the participants again were presented with all CSs but never received a shock. The procedure was the same as the retention test. The first three trials were always the three different CS types in an order that was counterbalanced across subjects.

### Physiological measures

Electrodermal activity (EDA) and heart rate were measured throughout the experiment (5000 Hz). EDA was measured via two Ag/AgCl electrodes attached to distal phalanges of the first and second finger of the right hand. Heart rate was measured via a pulse sensor attached to the third finger of the right hand However, this measure was collected for the purpose of a different study on individual differences in threat acquisition (results not shown here). Additionally, a woollen gauntlet was pulled over the participant’s right hand in order to keep the hand warm during the experiment.

### Subjective measures

Subjective ratings of shock expectancy and stimulus likeability were measured once at the end of day one and day three. We used a five-point rating scale, where the value of one corresponded to “*Not at all*” and the value of five corresponded to “*Very much*”. All stimuli were shown consecutively and in a randomised order on the screen with a self-paced, mouse-operated rating scale below. In order to gauge shock expectancy, the question “*How much do you expect a shock to be paired with this stimulus?*” was shown above the stimuli. Likability was then measured with the question “*How much do you like this stimulus?*”. Furthermore, we also measured trait anxiety with the State-Trait Anxiety Inventory^52^ and early-life adversity with the Childhood Trauma Questionnaire^53^, however these measures were also collected for the purpose of a different study on individual differences in threat acquisition (results not shown here).

### 2-back working memory task

In order to interfere with the reconsolidation process after memory reactivation, participants completed 20 min of a 2-back working memory task^30^. This task consists of successive presentations of different numbers where it is the participant’s task to indicate whether or not the number currently on the screen is the same as the number two trials back. Each number was presented for 0.4 s, followed by a 1.1 s ITI depicting a fixation cross. The participants were instructed to press the space bar when the number on the screen was the same one as the number two trials back. They could respond during the stimulus presentation or during the subsequent fixation cross and were notified on the screen whenever they pressed the space bar. The participants however did not receive any feedback on their performance. After 15 stimulus presentations the participants got a ten seconds break. There were 37 blocks resulting in a ~ 20 min duration in total.

## Data-analyses

After down sampling (200 Hz), raw skin conductance responses (SCR) data were scored with Autonomate^54^ implemented in Matlab^55^. Here, the amplitude of a rise in SCR was scored. The rise had to start between 0.5 s after stimulus onset and 0.5 s after stimulus offset, with a minimum rise time of 0.5 s and a maximum rise time of 5 s after response onset. Reinforced trials were omitted from the analyses. The SCRs from the acquisition phase were normalised to the average shock SCRs during the acquisition phase and square root transformed. The retention and retention after reinstatement SCR data were normalised to the average shock SCR during reinstatement and square root transformed. Even though stated otherwise in the preregistration, SCRs with z-scores above 3 or below -3 were not excluded from the analyses. Omitting this procedure had no consequences on the outcome and conclusion. The data were analysed with Bayesian Mixed Effects Models in R^56^ using the *brms* package^57^. Post-hoc contrasts were done with the package *emmeans*^58^ . Acquisition SCR data was analysed with a model including CS type (CS + , CS + r, CS-) as a fixed effect and a random intercept for Participant IDs with a random slope for CS type.

In order to test within-subject differences in SCRs across the different CSs during retention, we ran three models for each retention test separately (Retention, Retention after reinstatement). All models included CS type (CS + , CS + R, CS-) as a fixed effect and a random intercept for Participant IDs with a random slope for CS type. In addition, the second model included Time (First half, Second half) as a fixed effect and as a random slope across participants to account for the possible occurrence of extinction. In the last model, we compared the last trial of the acquisition phase (or retention test phase) with the first trial of the retention test phase (or retention after reinstatement test phase), with a similar random effect structure as above, only now with Phase (Acquisition, Retention, Retention after reinstatement) as a fixed effect and random slope. Since we used dummy coding for the stimuli here and were interested in contrasts between all CS type*Phase interactions, we ran two separate models with different dummy coding for the stimuli. Even though we did not preregister it at the time, we opted to add a fixed effect indicating which CS type was shown first during retention and retention after reinstatement. This allowed us to control for possible primacy effects.

Likeability and Expectancy rating data were analysed with a model including CS type (CS + , CS + r, CS-) and Day (Day one vs. Day three) as fixed effects, and a random intercept for Participant ID with a random slope for the CS type*Day interaction. In order to test for differences in Likeability and Expectancy ratings across the CS types, we ran a model with the respective subjective rating as the dependant variable, CS type as the fixed effect and as a random slope across participants. We then compared post-hoc contrasts.

Since Bayesian analyses do not yield p-values but instead work with 95% confidence intervals (CI), effects were considered “significant” in the traditional sense when the 95% confidence interval of the posterior distribution did not include zero. In addition to the confidence interval, we also report the estimate. The estimate is the mean of the posterior distribution, which is the probability distribution of the parameters conditional on the data.

## Results

### Skin conductance responses during acquisition

We first tested whether our threat-conditioning paradigm elicited conditioned responses during Acquisition. Please note that participants who did not show a numerically higher response to both CS + s compared to the CS − during acquisition were excluded from all analysis (see “Participants" of the Methods section for exclusion criteria).

**Figure 2:**
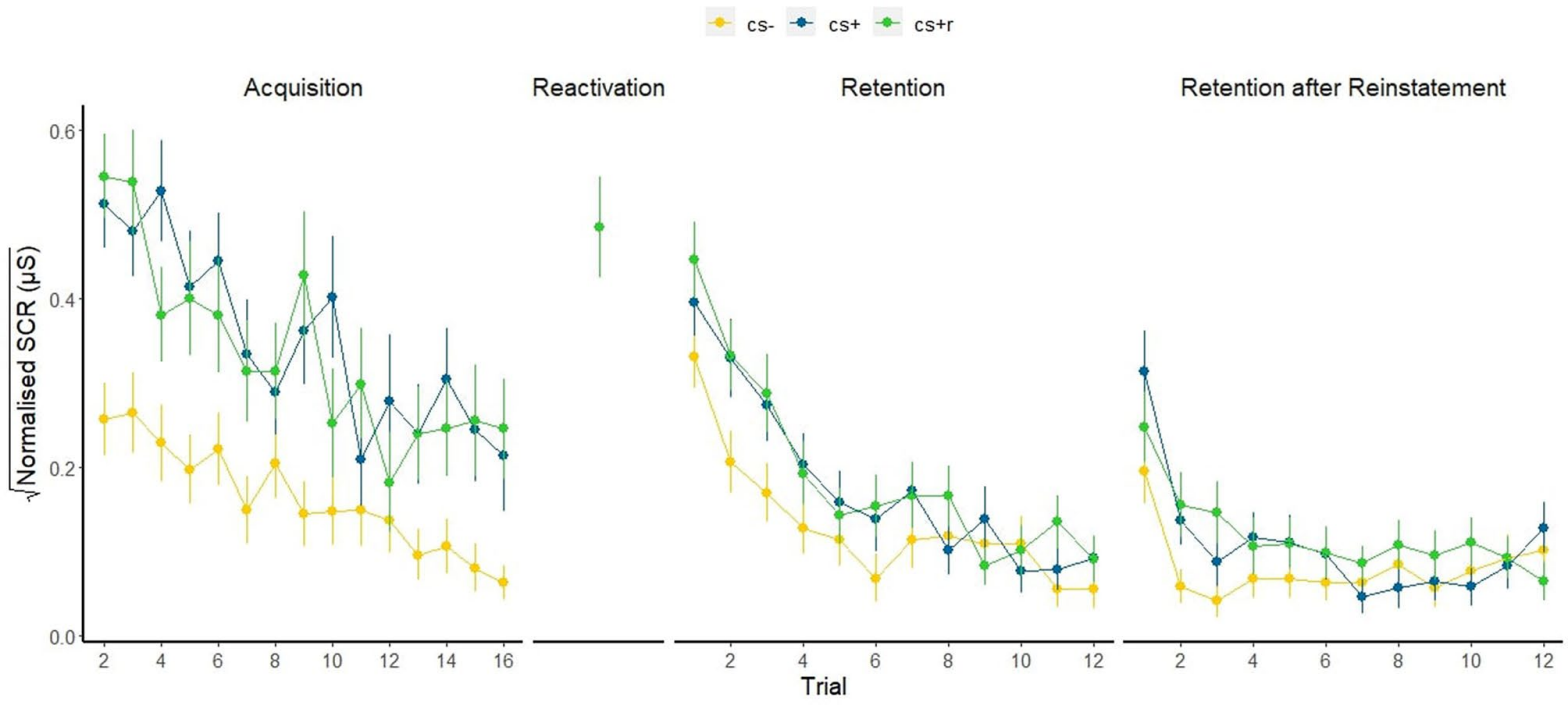
Time course of SCRs in response to the CS + R, CS + , CS- across all trials an all phases. Error bars indicate standard error of the mean. CS = conditioned stimulus, R = reactivated CS. This figure was created using the ggplot 2^70^ package in R^56^.

Our results show a significant difference in SCRs between the CS + and the CS- (estimate = − 0.19, 95% CI [− 0.24; − 0.15]) and between the CS + R and the CS- (estimate = − 0.16, 95% CI [− 0.19; − 0.11]), but there was no significant difference between the CS + R and the CS + (estimate = − 0.04, 95% CI [0.01; − 0.07]). Thus, we found successful acquisition of conditioned threat. See Fig. 2.

### Skin conductance responses during the Retention phase

Next we tested our primary hypothesis, namely that performing a 2-back working memory task after memory reactivation would interfere with the reconsolidation of the conditioned memory leading to a reduced conditioned response during Retention. First, across all trials of the Retention test phase, we found that SCRs to CS + (estimate = − 0.05, 95% CI [− 0.08; − 0.02]) and CS + R (estimate = − 0.06, 95% CI [− 0.1; − 0.02]) were significantly higher compared to SCRs to the CS-, indicating evidence of retention. However, in contrast to what we expected, SCRs to the CS + R and the CS + did not significantly differ from each other (estimate = − 0.01, 95% CI [− 0.03; 0.01]).

Second, we compared SCRs to the CS types across the First and Second half of the retention test separately. In the First half, SCRs to the CS + (estimate = − 0.08, 95% CI [− 0.13; − 0.04]) and CS + R (estimate = − 0.09, 95% CI [− 0.13; − 0.05]) were significantly higher compared to SCRs to the CS− , but this was not the case anymore during the Second half (CS + : estimate = − 0.02, 95% CI [− 0.06; 0.01], CS + R: estimate = − 0.04, 95% CI [− 0.08; 0.01]) indicating evidence of extinction. However, the CS + and CS + R did not differ significantly from each other during the First half (estimate = − 0.01, 95% CI [− 0.04; 0.02]) which remained non− significant during the Second half (estimate = − 0.01, 95% CI [− 0.04; 0.01]).

Last, we also did not observe significant differences in SCRs from the last trial of Acquisition to the first trial of the Retention phase between the CS + R and the CS + (estimate = − 0.04, 95% CI [− 0.12; 0.19]), as indicated by the absence of an interaction. Here, for the CS− , SCRs from the last trial of Acquisition to the first trial of the Retention phase also did not significantly differ from CS + R (estimate = 0.07, 95% CI [− 0.06; 0.21]) and CS + (estimate = 0.02, 95% CI [− 0.20; 0.07]). There was a main effect of Study Part (estimate = 0.21, 95% CI [0.11; 0.31]), indicating that SCRs for all CS types increased from acquisition to retention test. However, Rhats for this analysis were above 1.00, indicating problems with convergence. Results should be interpreted with caution. Together these three analyses approaches indicate that retention was similar for the CS + and CS + R relative to the CS-. See Fig. 2.

### Skin conductance responses during the retention after reinstatement phase

Next we tested, in a similar fashion to the retention phase, whether performing a 2-back working memory task after memory reactivation would lead to a reduced conditioned response during Retention after reinstatement.

First, across all trials of the Retention after reinstatement phase, we found that SCRs to the CS + (estimate = − 0.03, 95% CI [− 0.05; − 0.0007]) and CS + R (estimate = − 0.03, 95% CI [− 0.06; − 0.01]) were significantly higher compared to SCRs to the CS-. Again, in contrast to what we expected, SCRs to the CS + and the CS + R did not significantly differ from each other (estimate = − 0.01, 95% CI [− 0.03; 0.01]).

Second, in the First half, SCRs to the CS + (estimate = − 0.07, 95% CI [− 0.10; − 0.03]) and CS + R (estimate = − 0.07, 95% CI [− 0.10; − 0.03]) were significant higher compared to SCRs to the CS-, but this was not the case anymore during the Second half (CS + : estimate = 0.00, 95% CI [− 0.03; 0.03], CS + R: estimate = − 0.02, 95% CI [− 0.04; 0.01]) indicating evidence of extinction. However, the CS + and CS + R did not differ significantly from each other during the First half (estimate = 0.00, 95% CI [− 0.0.3; 0.03]) which remained non-significant during the Second half (estimate = − 0.02, 95% CI [− 0.05; 0.02]).

Last, the interaction analysis between CS type and Phase revealed that there were no significant differences in SCRs from the last trial of the Retention test phase to the first trial of the Retention after reinstatement phase between the CS + R and the CS + (estimate = − 0.0.7, 95% CI [− 0.05; 0.2]). Here, for the CS-, SCRs from the last trial of the Retention test phase to the first trial of the Retention after reinstatement phase also did not significantly differ from (estimate = 0.12, 95% CI [− 0.03; 0.26]) and CS + (estimate = 0.05, 95% CI [− 0.05; 0.17]) as indicated by the absence of an interaction. There was a main effect of study part (estimate = 0.17, 95% CI [0.08; 0.26]), indicating that SCRs to all CS types did significantly increase from Retention to Retention after reinstatement.

Together and in line with the results from the Retention test, these three analyses approaches indicate that also Retention after reinstatement was similar for the CS + and CS + R relative to the CS-. See Fig. 2.

### Subjective ratings of valence and shock expectancy during acquisition and retention

Next, we tested whether performing a 2-back working memory task after memory reactivation would lead to a reduction in subjective ratings of stimulus likeability and shock expectancy.

After the acquisition phase, the likeability ratings for the CS + (estimate = 2.24, 95% CI [1.79; 2.70]) and the CS + R (estimate = 1.90, 95% CI [1.31; 2.41]) were significantly lower compared to the CS-. Moreover, likeability ratings for the CS + and CS + R did not differ significantly from each other (estimate = − 0.34, 95% CI [− 0.77; 0.09]). We found a similar pattern with regards to the shock expectancy. Namely, shock expectancy ratings for the CS + (estimate = − 0.2.63, 95% CI [− 2.94; − 2.33]) and the CS + R (estimate = − 2.85, 95% CI [− 3.14; − 2.57]) were higher compared to the CS-. However, shock expectancy between the CS + and CS + R (estimate = − 0.22, 95% CI [− 0.06; 0.02]) did not significantly differ. Thus, the CS + and CS + R were equally disliked after acquisition and participants rated the likelihood that the CS + or CS + R were paired with a shock similarly.

While after Retention after reinstatement, at the end of day 3, the likeability for CS + (estimate = 1.27, 95% CI [0.79; 1.75]) and the CS + R (estimate = 1.01, 95% CI [0.41; 1.52]) remained to be rated lower compared to the CS-, again the CS + and CS + R did not differ significantly from each other (estimate = − 0.27, 95% CI [− 0.68; 0.18]). Shock expectancy ratings for the CS + (estimate = − 1.05, 95% CI [− 1.42; − 0.63]) and the CS + R (estimate = 1.22, 95% CI [− 1.58; − 0.86]) were still significantly higher compared to the CS- at the end of day 3 after both retention phases had occurred and did not differ significantly from each other (estimate = − 0.17, 95% CI [− 0.46; 0.11]).

**Figure 3:**
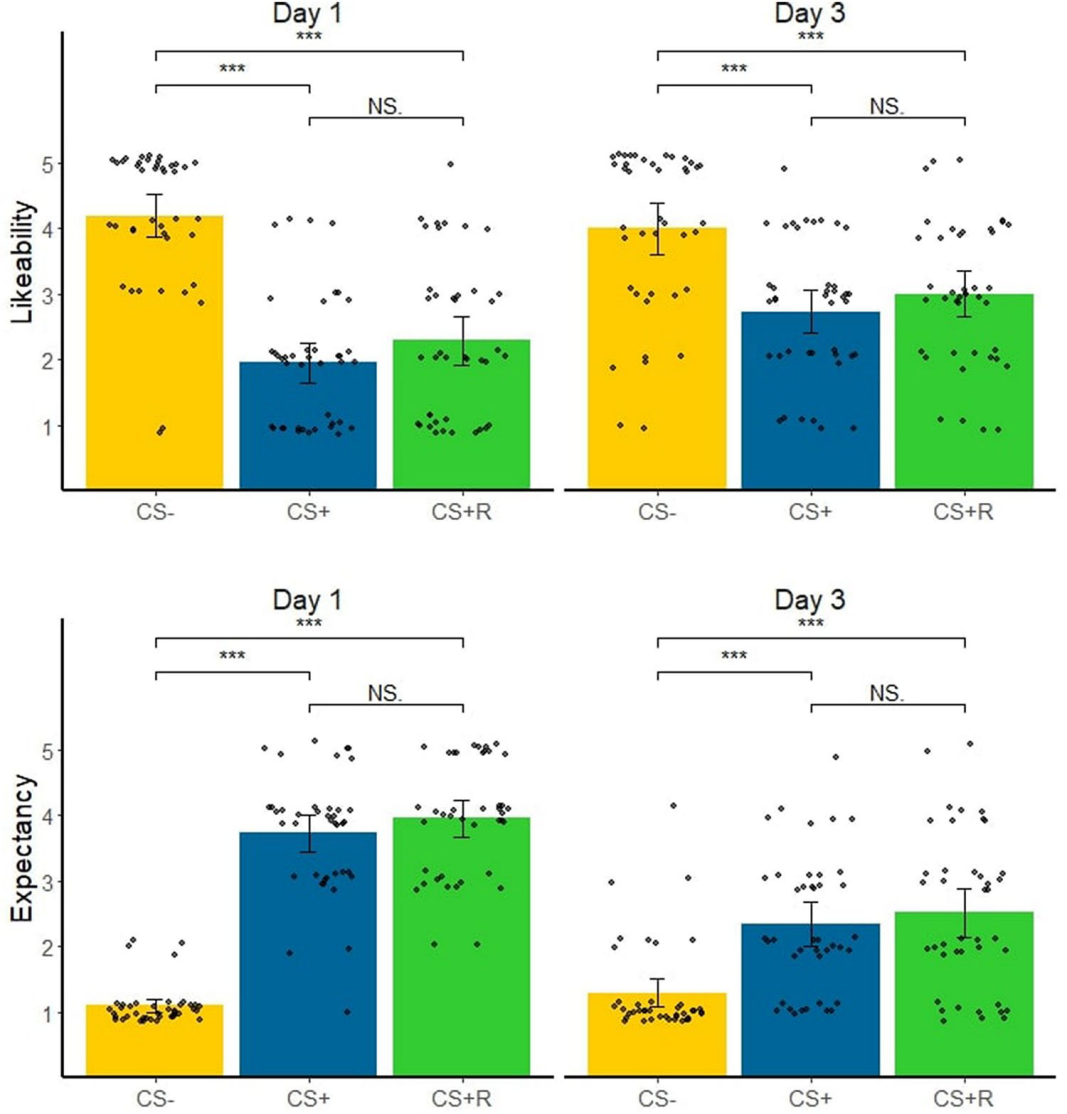
Likeability and expectancy ratings at the end of day 1 and at the end of day 3 for the three stimulus types. This figure was created using the ggplot 2^70^ package in R^56^.

Thus, in line with the analysis on the SCRs, we did not find evidence that performing the 2-back working memory task after memory reactivation changed the subjective stimulus likeability nor expectancy ratings of the reactivated conditioned stimulus. See Fig. 3.

### 2-Back task descriptive Statistics

Lastly, we verified whether participants performed the 2-back working memory task correctly and explore whether individual differences in performance would be related to individual differences in retention.

**Figure 4:**
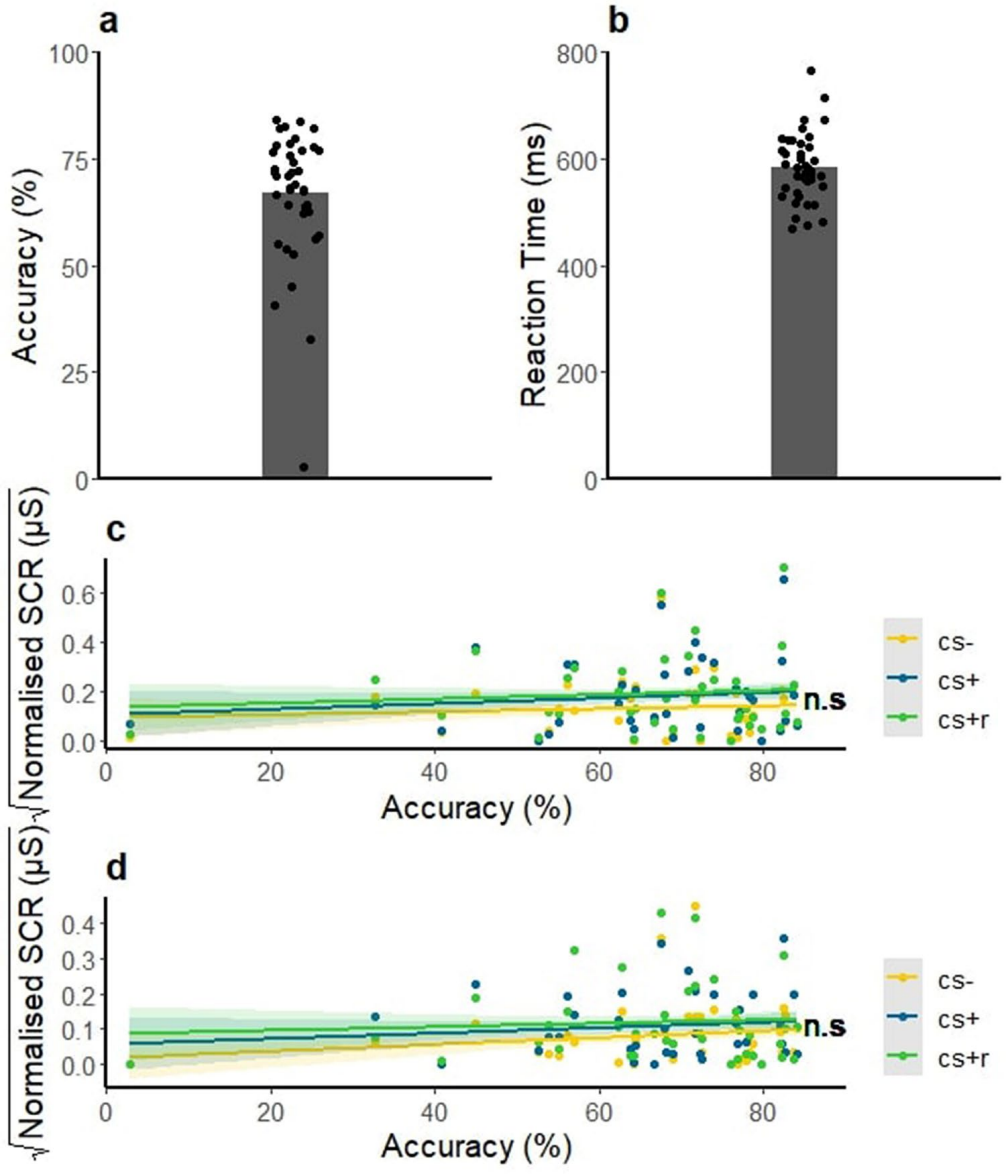
(**A**) Accuracy (hit rate minus false alarm rate) for the correct 2-back trials. (**B**) Reaction time in ms for the correct 2-back trials. (**C**) SCRs to all three CS types during Retention by Accuracy (hit rate minus false alarm rate) for the correct 2-back trials. Coloured shading indicates standard error of the mean. Plot D: Same as (**C**), but during Retention after reinstatement. (**C**) and (**D**) show that working memory performance does not seem to alter the way participants react to each stimulus. This figure was created using the ggplot 2^70^ package in R^56^.

Overall Accuracy (hit rate minus false alarm rate) was 66% (*SD* = 15%). Overall Reaction time in response to correct 2-back trials was 570 ms (*SD* = 60 ms). In order to test whether individual differences in SCRs between CS + and CS + R were correlated to individual differences in Accuracy, we correlated the difference score between CS + and CS + R during the Retention test and Retention test after reinstatement with the Accuracy on the 2-back working memory task. Pearson’s product moment correlations, however, show a non-significant correlation between Accuracy and SCRs in the Retention test phase (*r* = − 0.05, *t* = − 0.36, *df* = 39, *p* = 0.7178) as well as SCRs in the Retention test after reinstatement phase (*r* = − 0.10, *t* = 0.65, *df* = 39, *p* = 0.517). Thus, participants were able to perform the 2-back working memory task, but individual difference in performance could not account for individual difference in retention. See Fig. 4.

## Discussion

In this study, we aimed at assessing whether a cognitively demanding 2-back working memory task following memory reactivation could disrupt the reconsolidation of conditioned threat memories. Our threat conditioning paradigm was successful in eliciting acquisition and retention of conditioned threat indicated by increased SCRs to both the CS + and CS + R compared to the CS-. However, the reactivation procedure and 2-back working memory intervention did not lead to a significant difference in retention during both the retention phase and retention after reinstatement phase. Furthermore, after retention at the end of day 3, participants rated both CS + and CS + R similarly as less likable compared to the CS- and with a similar shock expectancy that was higher compared to the CS-. This indicates that in addition to psychophysiological conditioned threat responses, subjective feelings regarding the two CS + s were not altered after a memory reactivation and 2-back working memory task intervention. Therefore, we did not find evidence that the cognitively demanding working memory intervention can interfere with the reconsolidation process of threat memories.

Our findings seem in contrast to a large body of literature that has consistently shown that reactivation of aversive memories together with the execution of a cognitively demanding intervention can alter reactivated memories such that they become less aversive when retrieved later (for a meta-analysis see^1^). These studies have shown reductions in subjectively perceived emotionality and/or vividness of the memory when retrieved later^25,26^, reductions in physiological measures such as startle^23^, and reductions in intrusive memories^59,60^. Importantly, an often-used interpretation of these collective findings is the alteration of the reconsolidation process of the retrieved memory. However, a likely crucial difference between those study designs and ours is that in these experiments the cognitively demanding intervention was *co-occurring* (e.g.^1,22–26^) or performed immediately after *repeated* memory reactivation (e.g. ^29,30,44^). In contrast to that, our study employed a single reactivation trial in order to trigger a reconsolidation process instead of extinction^27^, as well as a 10 min wait period in order to accommodate memory destabilisation to take place before the intervention is started^16–19^ which is a procedural step in line with various other studies^32,33,35,46,51^. While the conceptual idea of these studies appears to be comparable (i.e. altering the experience of retrieved aversive memoires via cognitively demanding tasks), the procedural differences between the paradigms could lead to the involvement of altogether different memory processes. As these studies forego some of the necessary procedural steps (single reactivation, timing of the intervention) necessary to trigger reconsolidation, it is possible the beneficial effects of the cognitively demanding intervention cannot be attributed to reconsolidation interference.

Indeed, when including a single reactivation trial and performing a cognitively demanding intervention within the reconsolidation window, we do not find a disruption of the reconsolidation process of conditioned threat memories. Interestingly, this finding is in line with a previous study^35^ that also reported an absence of an effect of performing a working memory task following memory reaction on retention. Even though their design differs from ours in a few ways, our conclusions are comparable. For example, they employed an emotional working memory task instead of a non-emotional working memory task that we used in our design. Moreover, they made use of a between-subjects design while we used a within-subject design. Therefore, despite differences in study design, the outcomes of our study and the study conducted by Chalkia and colleagues^35^ are similar, implying generalisability across different designs. However, see a recent study which showed disrupted reconsolidation of conditioned threat memories by combining a between subject design with a non-emotional working memory task^45^. It is therefore possible that procedural design choices like these can also play an important role in triggering and altering the reconsolidation process. In sum, our study contributes to converging evidence indicating the importance of procedural steps in reconsolidation studies, thereby highlighting the difficulties of manipulating the reconsolidation process.

Nevertheless, a few studies have reported a reduction in intrusive memories of aversive movie clips due to playing a computer game of Tetris following memory reactivation of the movie^32,33^ when including the above mentioned procedural steps to trigger reconsolidation. How can this seeming discrepancy between our findings and the findings in these studies be interpreted? These studies, as well as the present one, all include aversive memories as the target memories, a memory reactivation procedure designed to destabilise the memory (as opposed to trigger an extinction process), and a cognitively demanding intervention^61^ during the reconsolidation window, both of which may party involve similar neural pathways^42^. While the nature of the aversive memory differs between both types of studies (audio-visual semantic memory and implicit memory)—and therefore also their assessment – these studies have the same aim: Disrupting reconsolidation of aversive memories with a cognitively demanding intervention. Therefore, the studies are conceptually comparable. One possibility for the discrepancy is that that the disruption of the reconsolidation process of aversive memories with a working memory intervention may be specific to certain aspects of the aversive memory and not others. Indeed, both James et al.^32^ and Kessler et al.^33^ have only reported a reduction in intrusive memories, but declarative recognition memory for the aversive movie clips was still intact. If this is the case, it raises important questions regarding the mechanism underlying the reconsolidation process of aversive memories and the possibility to interfere with it.

If cognitively demanding interventions, such as the 2-back working memory task we employed here, do not have the potential to interfere with reconsolidation, how can the previous beneficial effects on the reduction of aversive memories^1,22–26,32,33^ be explained? One possible interpretation is that through repeated memory reactivation, extinction is induced and thus the cognitively demanding intervention could interfere with extinction^29,30,43,62^. It has previously been shown that cognitively demanding interventions lead to amygdala inhibition^42,43^ and it has been argued that such additional inhibition of the amygdala during extinction could strengthen the extinction memory^29,30,42^. Another possibility is that cognitively demanding interventions may lead to a devaluation of the US. It was found that when participants were instructed to imagine the US and simultaneously made goal-directed eye movements, this led to a reduction in the conditioned response to the CS during the retention test^63^. However, in contrast to the previous study, in our design the US was not manipulated. Indeed, as both possibilities could not have occurred in our current experiment, it may be a possible explanation for why in our design the cognitively demanding intervention did not lead to altered conditioned threat responses. These are important considerations; as such interventions can only be optimized when fully understanding its underlying mechanism.

In conclusion, we did not find evidence for the notion that a cognitively demanding intervention alters reconsolidation of conditioned threat memories. A growing body of literature shows that the reconsolidation process of aversive memories is an intricate and delicate process that cannot be easily manipulated with behavioural interventions in humans^35,51,64–68^. While it is possible that cognitively demanding interventions may disrupt the reconsolidation process of aversive memories with respect to intrusive memories^32,33^, the failure of targeting the reconsolidation of threat memories (^35^ and the present study) puts to question the mechanism and its clinical applicability^69^. It is imperative that the working mechanism of therapeutic interventions should be understood, in order to optimise these interventions in the future. Our findings may have implications for the interpretation how cognitively demanding interventions could enhance psychotherapy and call for caution with using reconsolidation as an explanatory mechanism through which cognitively demanding interventions attenuate aversive memories.

## Data Availability

The data sets that support the findings of the current study and the code used to generate them are available in the Donders Institute repository: https://doi.org/10.34973/7baf-9q29
